# Supplementary material for: Antibiotics promote susceptibility to C. difficile infection through a CCR5-dependent immune response
Source: bioRxiv. 2026 Jun 29:2026.06.24.734321. Preprint. [Version 1] doi: 10.64898/2026.06.24.734321 (PMC13345209; doi:10.64898/2026.06.24.734321)
Supplement: Supplement 1 [file NIHPP2026.06.24.734321v1-supplement-1.pdf]

bioRxiv preprint doi: https://doi.org/10.64898/2026.06.24.734321; this version posted June 29, 2026. The copyright holder for this preprint (which was not certified by peer review) is the author/funder, who has granted bioRxiv a license to display the preprint in perpetuity. It is made available under a CC-BY 4.0 International license.

Supplementary Fig. 1 Flow cytometry gating strategy of the mouse colon

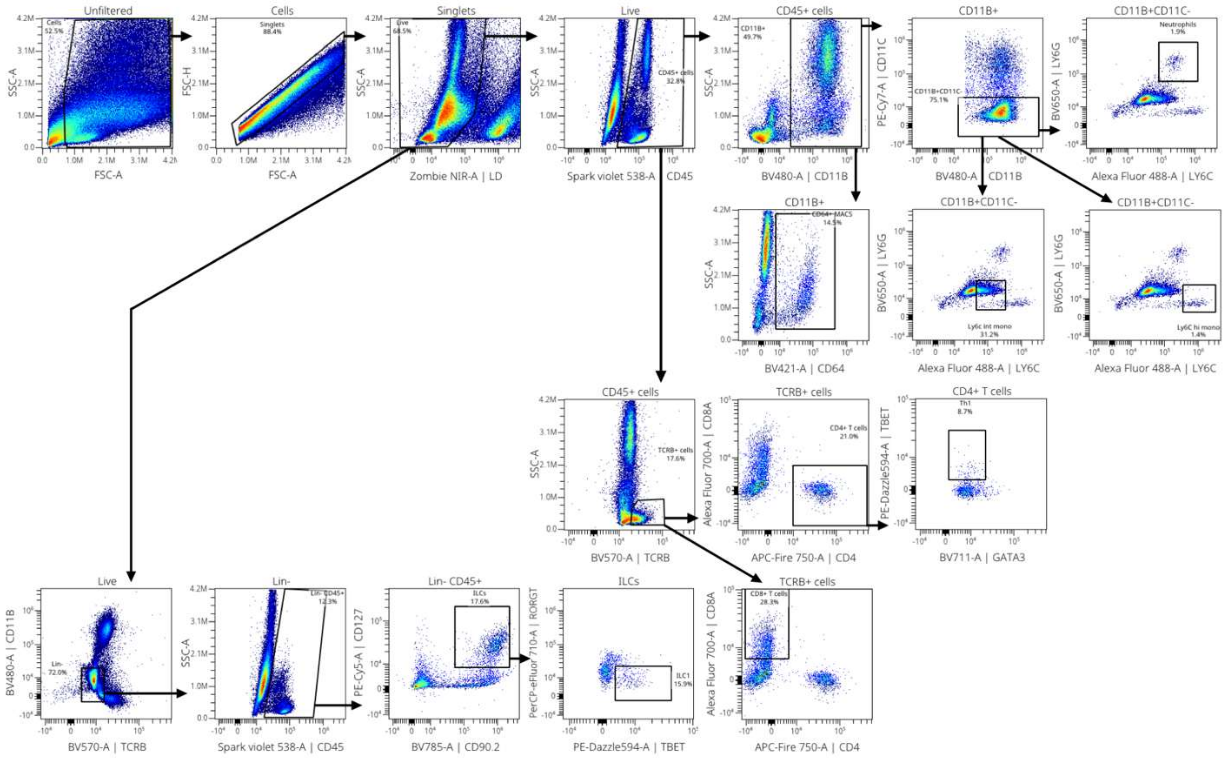

A representative flow cytometry gating strategy of a colon from a C57BL/6J mouse. Following SSC-A/FSC-A filtering and doublet exclusion pre-gating, Live CD45$^+$ cells were split into TCRβ$^+$ and CD11b$^+$ gates to subsequently enumerate CD4$^+$ T cells, CD8$^+$ T cells, Ly6C^{int} monocytes, Ly6C^{hi} monocytes, and neutrophil populations. The CD4$^+$ T cell pool was further divided according to TBET and GATA3 expression. To quantify ILC subpopulations, live CD45$^+$ lineage-negative (Lin$^-$; CD11b$^-$ TCRβ$^-$) cells were gated on CD127/CD90.2 and further segmented according to TBET and RORγT expression.

bioRxiv preprint doi: https://doi.org/10.64898/2026.06.24.734321; this version posted June 29, 2026. The copyright holder for this preprint (which was not certified by peer review) is the author/funder, who has granted bioRxiv a license to display the preprint in perpetuity. It is made available under a CC-BY 4.0 International license.

Supplementary Fig. 2 Colonic myeloid cell composition following ABX treatment

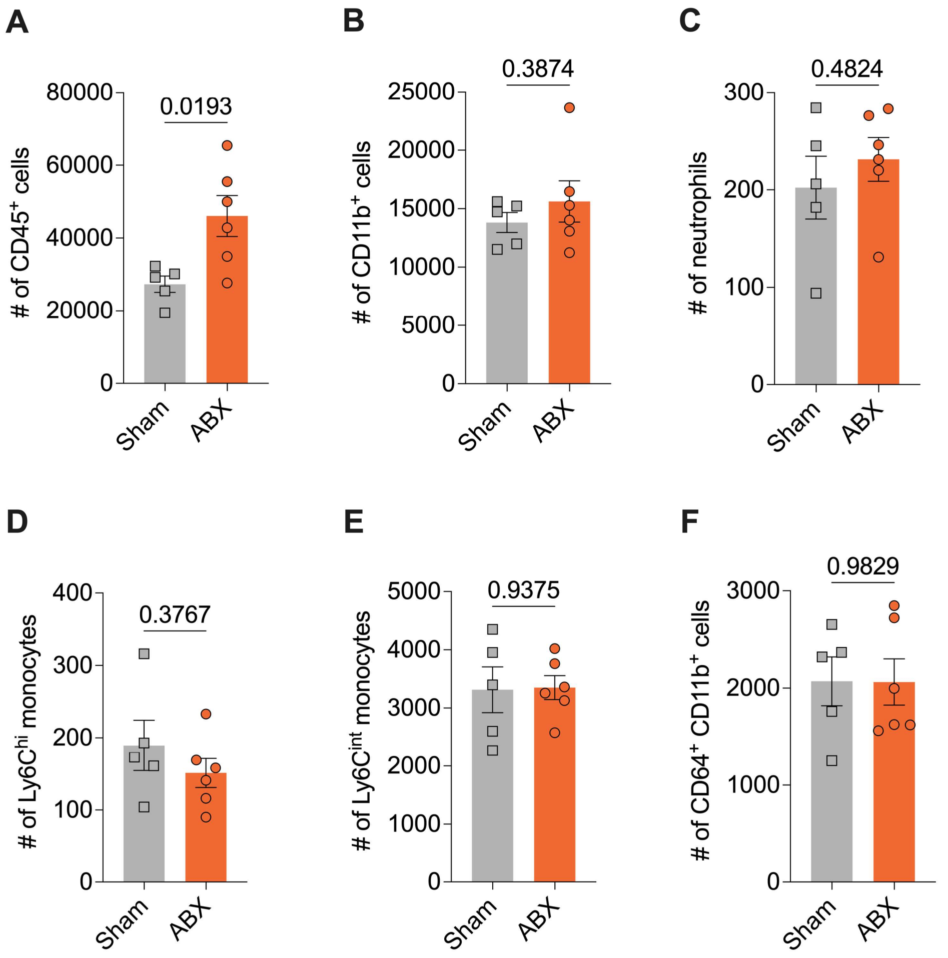

C57BL/6J mice received (1) either normal drinking water (sham treatment) or drinking water supplemented with an ABX cocktail of metronidazole, vancomycin, gentamicin, and colistin (ABX treatment) and (2) an i.p. injection of either a sham solution (sham treatment) or clindamycin (ABX treatment). The colon of each mouse was harvested 7 days after the start of the ABX regimen. (**A-F**) Cell count quantification of $CD45^+$ cells (A), $CD11b^+$ cells (B), neutrophils (C), $Ly6C^{hi}$ monocytes (D), $Ly6C^{int}$ monocytes (E), $CD64^+$ $CD11b^+$ cells (F) per harvested colon. Statistics calculated by a Welch's t-test. Data are presented as mean (± SEM). Each symbol represents a value from an individual animal.

bioRxiv preprint doi: https://doi.org/10.64898/2026.06.24.734321; this version posted June 29, 2026. The copyright holder for this preprint (which was not certified by peer review) is the author/funder, who has granted bioRxiv a license to display the preprint in perpetuity. It is made available under a CC-BY 4.0 International license.

Supplementary Fig. 3 Therapeutic CCR5 inhibition promotes colonic eosinophilia during CDI

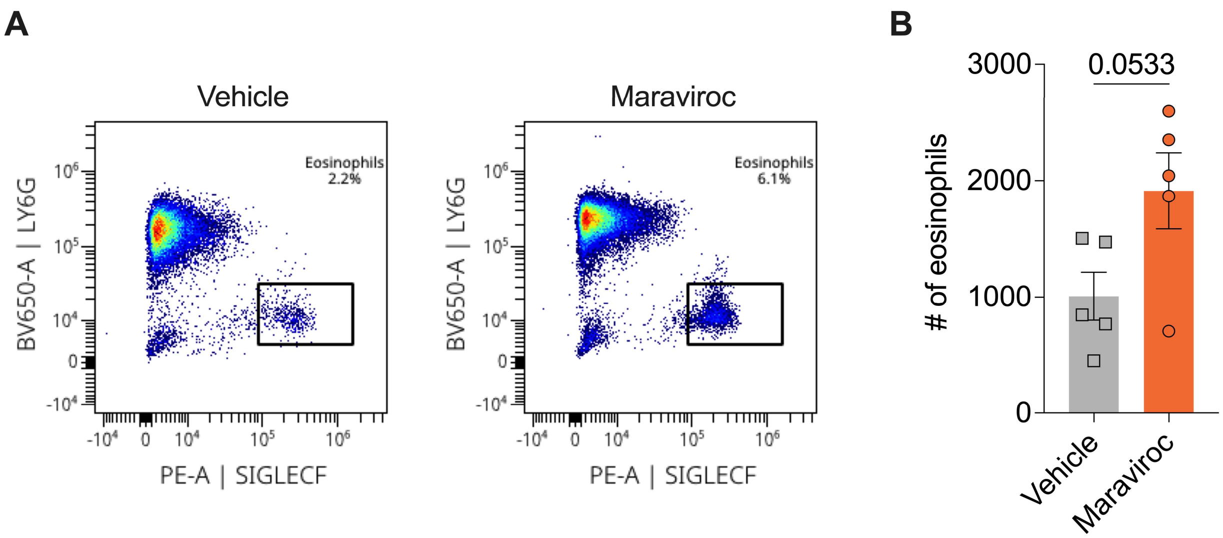

C57BL/6J mice received drinking water supplemented with an ABX cocktail of metronidazole, vancomycin, gentamicin, and colistin and an i.p. injection of clindamycin. Mice were infected with 1,000 *C. difficile* spores via oral gavage 24h after i.p. injection of clindamycin. On DPI -1, DPI 0, and DPI 1, mice were treated via i.p injections with either 1x PBS (vehicle) or a CCR5 inhibitor (maraviroc). The colon of each mouse was harvested on DPI 2 (7 days after the start of the ABX regimen). (**A**) Representative flow cytometry dot plots showing the gating strategy for eosinophils within the $CD11c^{-}$ $CD11b^{+}$ cell parent gate. (**B**) Cell count quantification of eosinophils per harvested colon. Statistics calculated by a Welch's t-test. Data are presented as mean (± SEM). Each symbol represents a value from an individual animal.
